# Amyloid-β, Tau, and α-Synuclein Protein Interactomes as Therapeutic Targets in Neurodegenerative Diseases

**DOI:** 10.1007/s10571-025-01604-7

**Published:** 2025-10-06

**Authors:** D. Mohan Kumar, Priti Talwar

**Affiliations:** Apoptosis and Cell Survival Research Laboratory, 412G Pearl Research Park, Department of Biosciences, School of Biosciences and Technology, Vellore Institute of Technology, Vellore, Tamil Nadu 632014 India

**Keywords:** Amyloid-β, Tau protein, α-synuclein, Protein interactomes, Therapeutic targets

## Abstract

Alzheimer’s and Parkinson’s disease are the most prevalent neurological diseases. Amyloid-β, tau, and α-synuclein proteins are known to be implicated in neurodegenerative disease (NDD). Elucidation of precise therapeutic targets remains a challenge. Therefore, the identification of interactomes of amyloid-β precursor protein (APP), microtubule-associated protein tau (MAPT), and α-synuclein (SNCA) proteins is of great interest, aimed at unraveling novel targets. An integrated analysis was employed to identify direct interactors as therapeutic targets, considering protein–protein interactions and subsequent network analysis. Further, it was proposed to identify hub proteins, intended targets, regulatory factors, disease-gene associations, functional enrichment analyses of the protein interactors interfered with gene ontologies and disease-driving pathways. Protein interactome centered on APP, MAPT, and SNCA identified the top hundred high-confidence protein–protein interactions that revealed BACE1, PSEN1, SORL1, GSK3B, CDK5, SNCAIP, PRKN, and APOE as physical and functional protein interactors. The top ten hub proteins were ranked based on multiple centrality measures and topological algorithms. Further, the integrated network of all three protein interactomes contained distinct nodes with edges. Interestingly, regulatory mechanisms have revealed possible regulatory modules, including cleavage, phosphorylation, and ubiquitination. Top interacting proteins were enriched in several ontology terms, such as regulation of neuronal apoptotic processes, amyloid beta fibril formation, and tau protein binding. Pathway analysis mapped the pathways of neurodegeneration-multiple disease, with a significant level of interacting proteins. Finally, the most comprehensive interactome associated with NDD provides insights into protein interactors, regulating the mechanisms of key proteins that can serve as novel therapeutic targets.

## Introduction

Neurodegenerative diseases like Alzheimer’s and Parkinson’s disease stand as the most prevalent diseases that affect millions of people globally, and the incidence of these diseases typically increases with aging. Neuropathological hallmark shared, along with many other neurodegenerative diseases (NDDs), is the aggregation of specific proteins within the brain (Goedert 2015; Compta and Revesz 2021). Alzheimer's disease (AD) is a widespread form of dementia worldwide, ultimately leading to profound cognitive and functional decline. AD is recognized by the accumulation of amyloidogenic-β peptides and microtubule tau proteins in the brain. According to the amyloid cascade, the buildup of β-amyloid peptides, specifically due to improper clearance, triggers a series of events that eventually result in disease. Amyloid-β is a small amphipathic peptide fragment originating from amyloid-β precursor protein (APP), a type of transmembrane protein found in neuronal synapses (Iliyasu et al. 2023). The tau hypothesis postulates that the aberrant tau protein accumulation as neurofibrillary tangles (NFT) in the brain is the primary contributory factor responsible for the progression of AD and its related tauopathies. Tau is encoded functionally by the microtubule-associated protein tau (MAPT) gene. Notably, tau becomes hyperphosphorylated, dissociates from microtubules, and aggregates to form intracellular neurofibrillary tangles (Guo et al. 2017). Conversely, Parkinson’s disease (PD) is a progressive neurological disorder that results in motor defects. PD is defined by intracellular deposits of α-synuclein aggregates, which are observed as a major component of Lewy’s, that are found to accumulate in dopaminergic neurons of the brain (Surguchov and Surguchev 2022). Notably, PD is the most common group of conditions known as synucleinopathy, which also includes multiple system atrophy. Alpha-synuclein, a natively unfolded protein derived from α-synuclein protein (SNCA), becomes misfolded and aggregates in cell inclusions to form intracellular Lewy neurites and Lewy bodies (Calabresi et al. 2023).

Amyloid-β (Aβ), tau, and α-synuclein (α-syn) are multi-disease-related proteins that work together across neurodegeneration-related diseases (Chen et al. 2023). These are the most studied protein aggregates that stand at the forefront of neurodegenerative pathology (Kumar and Kumar 2019). The protein pathogenesis of NDDs is related to a complex interplay of aging, genetics, environmental, and lifestyle factors. Assemblies of Aβ, tau, and α-syn proteins originate in specific brain regions and then spread in a predictable pattern to other areas of the brain (Haenig et al. 2020). The oligomeric buildups and fibrillar forms of the Aβ, tau, and α-syn drive synaptic loss, mitochondrial dysfunction, oxidative stress, intense neuronal inflammation, and neurotoxicity associated with progressive neuronal dysfunction and neuronal cell death (Calabrese et al. 2022). They are found to interact synergistically, promoting each other's aggregation and accumulation, which accelerates cognitive decline in AD and Lewy body dementia in PD (Clinton et al. 2010; Thompson et al. 2020). For instance, Aβ plaque deposits are noted to dramatically accelerate both the seeding and spreading of α-syn and tau aggregation in the brain (Bassil et al. 2020). The co-existence of these proteinopathies suggests that a comprehensive understanding of their interactome is crucial for developing targeted therapeutic interventions.

Development of therapeutic strategies to ameliorate pathological signs and prevent the worsening of symptoms is the main goal in managing NDD. An effective treatment for NDDs is still lacking, and it is imperative to gain deeper insight into disease pathophysiology is in urgent need to provide potential drug targets (Giacomelli et al. 2017; Yu et al. 2020; Sweeney et al. 2017). However, extensive research focused solely on APP/Aβ, MAPT/tau, and SNCA/α-syn neurotoxicity has shown limited efficacy in treating NDD. The interplay among these factors is undiscovered, and which pathways are the root causes of NDDs is under dispute (Yu et al. 2020). A novel approach now being explored is to identify the protein interactors that drive the accumulation of misfolded protein, aiming to prevent their prion-like propagation. A promising strategy targeting early protein processing stages like translation, cleavage, and post-translational modifications is essential in order to avoid misfolding and aggregation. This upstream approach prioritizes therapeutic specificity, eliminating toxic protein forms and preventing their propagation (Sweeney et al. 2017). Emerging studies suggest that the binding partners/interacting proteins, as well as the exact disease-driving key pathways linking Aβ, α-syn, and tau proteins to neurodegeneration, have yet to be elucidated through protein–protein interaction (PPI). For instance, it is unclear which protein interactors exacerbate protein aggregates in NDDs. This knowledge gap illustrates the necessity to investigate the triumvirate protein interactome, with an emphasis on their toxic effects (Sengupta and Kayed 2022).

An integrative study of the Aβ, α-syn, and tau interactome is a crucial step towards advancing NDD research. This approach finds key protein interactors that might serve as therapeutic targets for drug discovery, thereby offering a molecular foundation for predicting and modulating protein aggregation (Balasubramaniam et al. 2023). The disease mechanism of AD-PD can be extensively researched through investigating its protein–protein interactions, and an integrated strategy offers novel therapeutic avenues for the treatment of NDD (Snider et al. 2015; Spires-Jones et al. 2017; Basu et al. 2021). Elucidation of PPIs using a systems biology approach has further unraveled the protein interaction network (PIN), which provides additional insights about the roles of proteins implicated in NDD. Therefore, the main concern was to investigate the key drivers and molecular aspects of Aβ, tau, and α-syn, and targeting the protein-interacting partners responsible for toxic protein accumulation involved in the onset and progression of AD and PD may offer more therapeutic promise (Zhang et al. 2021a). The present research describes a systematic approach, focusing on neurodegeneration-related protein interactome mapping to discover disease-associated proteins as therapeutic targets.

## Materials and Methods

### Selection of NDD Protein

NDD-specific protein interaction network mapping starts with the identification of a central target protein. Major causative proteins for selected NDDs, particularly AD-PD, were identified and considered for further protein interaction analysis in order to identify key regulatory proteins. NDDs were driven by the pathological aggregation of specific causative proteins, involved in Alzheimer’s disease (APP and MAPT), and Parkinson’s disease (SNCA) (Ahmad et al. 2016).

### Identification of Interacting Proteins

A search tool for the retrieval of interacting genes and proteins (STRING) was employed for the identification of interacting proteins (Szklarczyk et al. 2023). The search was made to find the interacting protein partners of the APP, MAPT, and SNCA proteomes. STRING was utilized to retrieve the functional interactors by specifying a single protein as query input, and the organism was set to “*Homo sapiens*”. We applied STRING network type (functional and physical protein associations), evidence-based interactions, and also all active interaction sources, with high-confidence interactions and a combined score of 0.7 > high ≥ 0.9. A maximum of a hundred interactors in the first shell were selected for further analysis.

### Protein–Protein Interaction

STRING: The functional protein association networks tool was employed for the PPI study (Szklarczyk et al. 2023). The protein–protein interaction for APP, MAPT, and SNCA with its interacting protein partners was constructed from STRING, including both functional and physical associations. It compiles known interactions sourced from high-throughput experiments, with predicted interactions based on gene fusions, neighborhood, co-occurrence, protein homology, and co-expression analyses. The final combined scores of ≥ 0.9 are computed between the top pairs of proteins during interaction. PPI analysis defined interactions between proteins to predict specific insights into NDD. The PPI interaction was then imported into Cytoscape to construct the PPI network (Shannon et al. 2003).

### PPI Network Analysis

The STRING Analysis module was used to evaluate the PPI networks of APP, MAPT, and SNCA with multiple key metrics: including the number of nodes (interacting proteins), followed by the edges (protein–protein associations), average node degree, average local clustering coefficient, and the statistical significance of PPI enrichment value (*p* value) (Szklarczyk et al. 2023; Doncheva et al. 2019). Then, the Network Analyzer available in Cytoscape aids to compute and analyze a set of topological parameters, and specifically the key centrality measures such as node degree distribution and edge betweenness by degree in a PPI network (Assenov et al. 2008).

### Hub Protein Identification

The CytoHubba plugin in Cytoscape is a versatile tool for identifying hub genes/proteins and significant molecular interactions, offering a variety of ranking algorithms employed to assess various centrality measures of nodes, to rank the significance of each protein's interactions within their respective networks. The CytoHubba module was utilized to analyze the intricate interactions of APP protein interactors, MAPT interactors, and SNCA protein interactors by considering topology factors using local and global algorithms, such as degree, betweenness, and closeness centrality, which were selected to determine hub genes/proteins (Chin et al. 2014). Subsequently, the interconnectivity among these hub genes was analyzed and visualized with Cytoscape (Shannon et al. 2003).

### Network Integration

The PPI networks of APP, MAPT, and SNCA interactomes (each with 100 nodes) were first constructed separately, and these three networks were then integrated to get a single consensus network. These networks were integrated by concatenating interaction sources across PPI databases to retain high-confidence interactions (confidence score ≥ 0.7), thereby maintaining biologically significant interactions. The integrated network was processed and visualized using the force-directed layout in Gephi to identify network descriptors, such as nodes, edges, eigenvector centrality, and degree were utilized as inputs for independent variables for network analysis (Grandjean 2015). Node size was proportional to the degree (number of interactions), and edge thickness represented confidence scores. Integration of networks was crucial to uncover crosstalk between APP, MAPT, and SNCA, and subsequent analyses.

### Investigation of the Molecular Crosstalk

An interactive Venn analysis was employed to find cross-linking and unique protein interactors. The Venny tool was utilized for visualizing the overlap between different sets of protein interactors associated with Aβ, tau protein, and α-syn (Heberle et al. 2015). The retrieved top hundred interacting protein partners of APP, MAPT, and SNCA were selected and imported into the Venn dataset to find their overlapping and common potential interactors among these neurodegenerative disease-associated proteins. Thus, the shared interactors can pinpoint the key biological markers indicative of AD-PD crosstalk (Heberle et al. 2015).

### Human Protein Atlas

Human Protein Atlas (HPA) was utilized to retrieve the list of direct/physical interactors of APP, MAPT, and SNCA. The HPA consortium has brain resources for profiling human proteins (Uhlén et al. 2015). We were involved in searching for each protein and compiling the associated interactors. HPA provided access to the integrated protein–protein interaction resources derived from IntAct, BioGRID, BioPlex, and OpenCell that have been built with data related to protein expression, location, and classification. Thus, HPA was employed to assess interaction type, subcellular localization, cell type specificity, and protein class (Uhlén et al. 2015).

### Disease-Gene Association

DisGeNET provides an association between genes and human diseases. The DisGeNET web interface was accessed to retrieve the genes associated with NDD, particularly AD-PD. The search was refined to focus on associations with high DisGeNET scores, such as gene-disease association (GDA) score, which prioritizes genes ranging from 0 to 1; the closer the value to 1 is considered as most associated genes. Furthermore, the disease specificity index (DSI) value > 0.6 was assessed to articulate the specificity of the target genes/proteins relevant to a particular disease. The Association Type ontology dissects genes linked to the disease as biomarkers and therapeutic targets (Piñero et al. 2020).

### Regulatory Network

The SIGnaling Network Open Resource (SIGNOR) is an open-access repository that captures links between human proteins, phenotypes, stimuli, and biological significance. We employed advanced relation search on the SIGNOR platform, to retrieve the signaling relationship and role of regulators accompanied with a functional effect annotation (up/down-regulation) and together with the molecular mechanism (e.g. cleavage, phosphorylation, binding, transcriptional activation, etc.) on the regulation of the key target such as APP, MAPT, and SNCA protein entity. SIGNOR interactome was retained; for instance, only interactions were annotated to the human brain and the central nervous system (Surdo et al. 2023).

### Enrichment Studies

Functional enrichment analysis of the topmost interacting protein partners of APP, MAPT, and SNCA was performed with the help of DAVID. Further, Enrichr (integrated with multiple databases) is an intuitive web-based application for enrichment analysis that offers a variety of visual summaries of the collective functions of lists of gene-encoded proteins for the target organism (Xie et al. 2021). Subsequently, gene ontology enables the annotation of gene products and terms in a biological network module that are over-represented statistically among a set of proteins. Gene ontology analysis was done to discover significant terms organized into three main aspects: Biological Process (BP), Molecular Function (MF), and Cellular Component (CC) (Ashburner et al. 2000). The biological pathway assessment of top interactors was done using DAVID- Database for Annotation, Visualization and Integrated Discovery v6.8 web tool concerning the KEGG (Kyoto Encyclopedia of Genes and Genomes) database (Huang et al. 2009). The KEGG analysis was performed to identify possible functional pathways. The enriched terms were retrieved concerning FDR and P- adj. value of 0.05 (Kanehisa et al. 2025).

## Results

### NDD-Associated Proteins

APP, MAPT, and SNCA seed proteins were identified as key initiators of protein aggregates in AD-PD. Cross-link-based interactome analysis demonstrated the biological significance of their interactions in mediating protein misfolding cascades and pathological cross-seeding events. APP represents the primary protein implicated in the amyloid-cascade paradigm and the amyloidogenic process. MAPT is responsible for the tau deposition and NFT formation. SNCA drives the α-syn hypothesis, with Lewy’s aggregates. The AD part has extracellular β-amyloid plaque (abundance of Aβ42, an APP cleavage product) and intraneuronal NFT (hyperphosphorylated tau/MAPT fragments as paired helical filaments). PD is characterized by intraneuronal Lewy bodies (enriched in aggregated α-syn/SNCA).

### Interacting Partners of APP, MAPT, and SNCA

We retrieved a total of 100 high-confidence interactors for each of the three neurodegeneration-linked proteins: APP, MAPT, and SNCA in *Homo sapiens* (Supplementary material). The interacting partners of APP, MAPT, and SNCA were determined by a functional protein association tool called STRING. The top hundred protein interactors were identified and retrieved with high confidence, at the threshold of interaction score ≥ 0.7. APP-associated partners included amyloidogenic regulators such as PSEN1 and BACE1, inflammatory mediators like NFKB and TNF, and apoptotic proteins like CASP3/6. The MAPT interactome clustered around tubulin subunits and cytoskeletal dynamics (TUBB, MAP1B), kinases (CDK5, GSK3B, DYRKIA, and MAPK), and stress-response proteins (HSP90AA1). SNCA interactome highlighted protein clearance systems like NEDD4, CLU, and aggregation modifiers.

### Protein–Protein Interaction

PPI network was constructed for the protein interactors using the functional protein association network tool called STRING, with *Homo sapiens* specified as the target organism. The PPI networks for each protein comprised both direct (physical) and indirect (functional) associations, with APP, MAPT, and SNCA, as defined by STRING, exhibiting distinct interactomes. We selected and ranked the top 10 interacting partners among the hundred interactors of APP, MAPT, and SNCA with a confidence score of ≥ 0.9, which is directly connected with the target protein as provided in Table 1. The interactome of APP included notable interactors such as BACE1, APBB1, APOE, and PSEN1. Similarly, the MAPT interactome comprised key partners like GSK3B, CDK5, and Tubulin subunits like TUBA1B and TUBB2B. The interactors associated with SNCA, featuring important partners, are PRKN, PARK7, and Synphilin-1, encoded by the SNCAIP.

**Table 1:** PPI analysis of APP-amyloid-β, MAPT-tau, and SNCA-α-synuclein protein interactomes revealed the top interactors to gain specific insights into NDD

S. no.	APP interactors	Protein name	IntAct	STRING
1.	BACE1	Beta-site APP Cleaving Enzyme 1	Direct	0.999
2.	APOE	Apolipoprotein E4	Indirect	0.999
3.	APBB1	APP Binding Family B Member 1	Direct	0.999
4.	SORL1	Sortilin-Related Receptor 1	Direct	0.999
5.	PSEN1	Presenilin 1	Direct	0.999
6.	TNFRSF21	TNF Receptor Superfamily Member 21	Indirect	0.998
7.	NAE1	NEDD8 Activating Enzyme E1 Subunit 1	Indirect	0.998
8.	CLU	Clusterin	Indirect	0.998
9.	NCSTN	Nicastrin	Indirect	0.998
10.	APBA1	APP Binding Family A Member 1	Indirect	0.998

S. no.	MAPT interactors	Protein name	IntAct	STRING
11.	CDK5	Cyclin-Dependent Kinase 5	Indirect	0.999
12.	GSK3B	Glycogen Synthase Kinase-3 Beta	Direct	0.999
13.	APP	Amyloid Beta Precursor Protein	Direct	0.995
14.	TUBA1B	Tubulin Alpha 1B	Indirect	0.995
15.	SNCA	Synuclein Alpha	Direct	0.994
16.	FYN	Tyrosine-protein kinase Fyn	Direct	0.994
17.	TUBB2B	Tubulin Beta 2B	Indirect	0.994
18.	APOE	Apolipoprotein E	Indirect	0.991
19.	TUBB3	Tubulin Beta 3	Indirect	0.987
20.	TUBA4A	Tubulin Alpha 4A	Indirect	0.981

S. no.	SNCA interactors	Protein name	IntAct	STRING
21.	PRKN	Parkin RBR E3 Ubiquitin Protein Ligase	Indirect	0.999
22.	SNCAIP	Synuclein Alpha Interacting Protein	Direct	0.999
23.	TH	Tyrosine Hydroxylase	Indirect	0.997
24.	SLC6A3	Solute Carrier Family 6 Member 3	Indirect	0.996
25.	APOE	Apolipoprotein E	Direct	0.996
26.	CALM3	Calmodulin 3	Indirect	0.995
27.	TARDBP	TAR DNA-Binding Protein 43	Indirect	0.994
28.	PARK7	Parkinsonism Associated Deglycase 7	Indirect	0.994
29.	HSPA4	Heat Shock Protein Family A	Indirect	0.994
30.	MAPT	Microtubule-Associated Protein Tau	Direct	0.994

### PPI Network Analysis

The PPI networks of APP, MAPT, and SNCA each comprised 100 interactors but exhibited distinct topological properties, comprising 101 nodes (proteins) with varying degrees of edges (protein–protein association). We computed six network parameters of APP, MAPT, and SNCA interactomes by NetworkAnalyzer, which describes a protein node’s relationship. The APP interactome network contained 1142 edges, with a typical average node degree of 22.6, and reflected moderate modularity. The MAPT interactome network displayed 1543 edges, with the highest average node degree of 30.6. In contrast, the SNCA network featured 1308 edges, along with an average node degree of 25.9. All networks showed statistically significant PPI enrichment (*p* < 1.0e−16), confirming a random interaction clustering coefficient of 0.6, indicating a strong functional relationship among the interactors. The interactor with the highest node degree distribution represents the most valuable protein-coding target against NDD.

### Hub Proteins

The top 10 hub proteins within the interaction networks of APP, MAPT, and SNCA were identified based on four local rank techniques and six centrality measures. The top ten hub proteins were identified and ranked in a network by the average scoring of all the algorithms, such as Maximal Clique Centrality (MCC), Clustering Coefficient (CC), and the ratio of CC to Eccentrality (EC) > 0.5. All identified hubs exhibited a high degree ≥ 5, closeness ≥ 7, and betweenness centrality ≥ 0.5 (supplementary material). APBB1, PSEN1, and BACE1 were identified as top-scoring hub proteins in the APP interactome, with the highest degree (≥ 7) of betweenness centrality, among proteins with heightened connectivity. For the MAPT interactome, GSK3B and CDK5 were identified as top hub proteins, with heightened connectivity degree, followed by TUBA1B. In the SNCA network, PRKN, PARK7, and LRRK2 were found to be key hub proteins. Notably, all key parameters related to PPIN were computed to assess the significance of these hub proteins, with high scores.

### Integrated Network

**Fig. 1:**
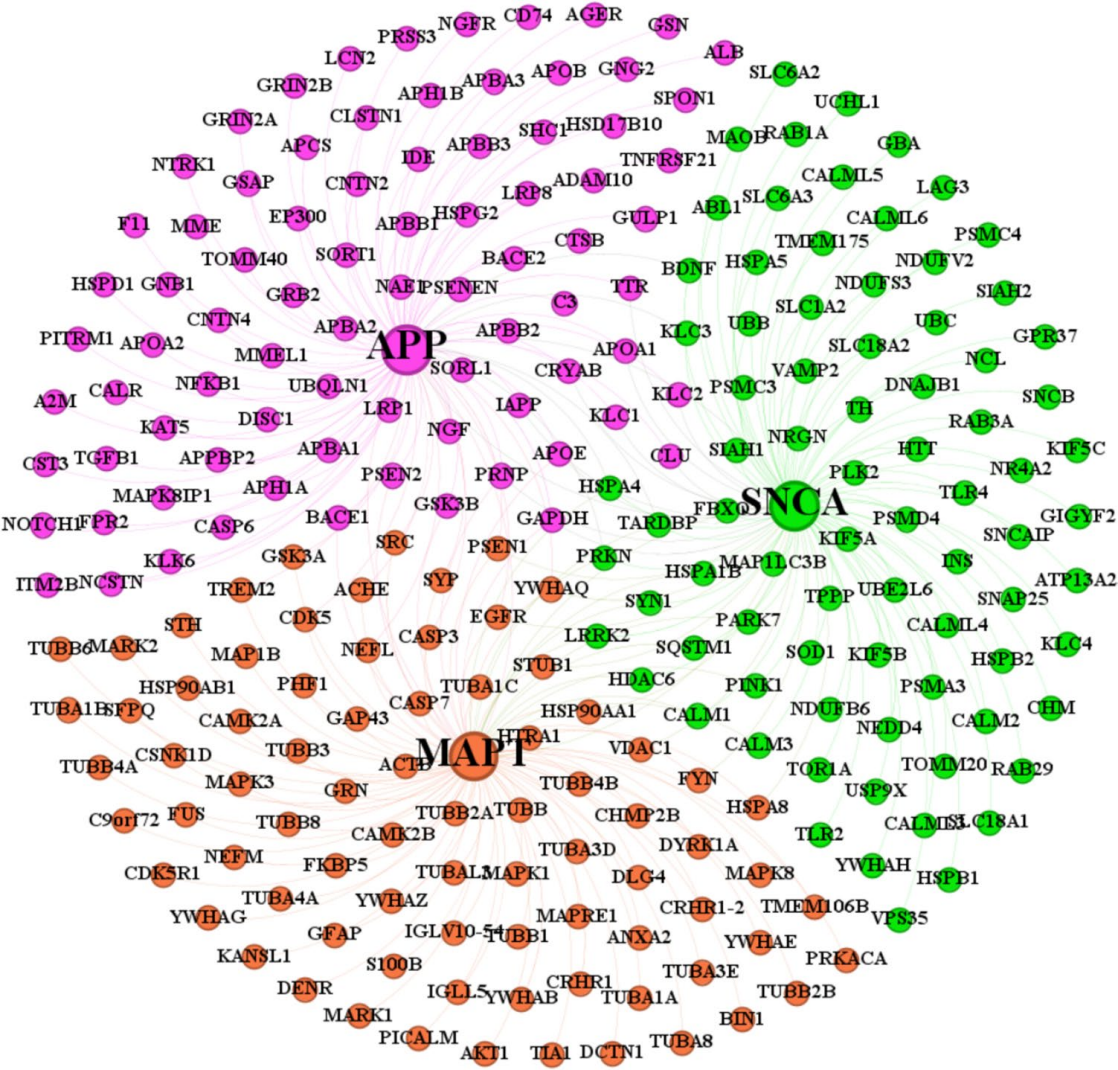
Integrated protein interactome of three key neurodegeneration-associated proteins: APP, MAPT, and SNCA. Each key protein is represented by a large central node with its respective 100 first-degree protein interactors shown as smaller nodes, color-coded by their association. The connecting edges represent physical interactions between proteins

The individual PPI networks of APP, MAPT, and SNCA were successfully integrated, each containing 100 nodes representing the most significant interacting partners with distinct topological properties. The final integrated consensus network has 3 central proteins that comprise distinct nodes with edges/interactions (Fig. 1), indicating substantial crosstalk between the three interactomes. The integrated network exhibited an intermediate density, reflecting the selective retention of high-confidence interactions during integration. Notably, the proteins were found to interact with all three proteins of interest (APP, MAPT, and SNCA), representing key mediators of crosstalk. An integrated interactome is depicted in the Fig. 1 provides a systems biology perspective on the molecular connections between these three critical proteins involved in neurodegeneration. Integrated network analysis revealed the complex interplay between APP, MAPT, and SNCA interactomes, highlighting shared molecular mechanisms that may contribute to the overlapping pathologies observed in NDD.

### Unique Interactors and AD-PD Crosstalk

The interactive Venn analysis investigated molecular crosstalk between AD and PD, revealing critical overlaps in protein interactors associated with Aβ (APP), Tau (MAPT), and α-syn (SNCA). Among the top 100 interacting partners of each protein network, 9 unique interactors, including APOE, PSEN1, PRNP, GAPDH, PRKN, HSPA4, TARDBP, EGFR, and YWHAQ, were identified as common across all three protein interactomes of Amyloid-β, tau, and α-syn. Further, Aβ and Tau protein interactomes shared 12 common interactors, while 10 interactors overlapped between Aβ and α-syn, and 15 interactors between Tau and α-syn (Supplementary material). These interactors provide insights into the molecular crosstalk driving AD-PD comorbidity and highlight potential therapeutic targets.

### Human Protein Atlas

HPA was utilized to compile direct/physical interactors of APP, MAPT, and SNCA by integrating PPI data from IntAct, BioGRID, BioPlex, and OpenCell, along with tissue-specificity and subcellular localization, listed in Table 2. BACE1, APBB1, and SORL1 directly interact with APP primarily in brain tissues, with cell specificity of oligodendrocytes, neurons, and microglial cells, localized to the endomembrane system. MAPT interacts directly with kinase GSK3B in the brain, showing cell specificity for neurons and oligodendrocyte precursor cells, with localization in the nucleus and intracellular compartments. Direct interactors of SNCA include SNCAIP and APOE in the brain tissues, particularly in astrocytes, neurons, oligodendrocyte precursor cells, and muller glia cells, localized in the cytoplasm and endomembrane system.

**Table 2:** Direct interactors of key proteins associated with NDD, along with their tissue and cell type specificity, subcellular localization, and protein classification

NDD protein	Direct interactors	Tissue specificity	Cell type specificity	Subcellular location	Protein class
APP	BACE1	Brain	Oligodendrocytes Photoreceptor cells (cone)	Endomembrane system Membrane, Intracellular	Enzyme/Transporter
	APBB1	Brain cerebellum	Oligodendrocytes	Endomembrane system Intracellular	Other
	SORL1	Brain	Neurons (Excitatory) Microglial cells	Non-specific Membrane, Secreted	Transporter
MAPT	GSK3B	CNS Brain	Neurons (Excitatory/Inhibitory) Oligodendrocyte precursor cell	Nucleus Membrane, Intracellular	Kinase
	FYN	Brain	Astrocytes Oligodendrocyte precursor cell	Endomembrane system Intracellular	Enzyme
SNCA	SNCAIP	Brain	Astrocytes Neurons (Inhibitory) Oligodendrocyte precursor cell	Cytoplasm Intracellular	Other
	APOE	Brain	Muller glia cells	Endomembrane system Secreted	Lipid-Transporter

### Disease-Gene Association

DisGeNET was analyzed, focusing on NDDs, particularly AD (CUI: C0002395), which integrates 5191 genes, and PD (CUI: C0030567), which integrates 3367 genes. We systematically retrieved only the top 100 protein coding genes found to be strongly associated with AD-PD by applying a high confidence DisGeNET GDA score at a threshold of ≥ 0.5 (range: 0–1), which was further refined using a DSI threshold > 0.6, resulting in high-specificity genes (Supplementary material) with prioritized roles in disease mechanisms. Notably, APP, MAPT, PSEN1, APOE, BACE1, ACHE, and TREM2 were strongly associated with AD. SNCA, LRRK2, PARKN, PINK1, PARK7, and MAOB were found to be strongly associated with PD. DisGeNET association type ontology of the AD-PD-associated gene was found to be altered expression, post-translational modification, causal/contributor, and biomarkers.

### Regulatory Mechanisms

SIGNOR analysis identified regulatory mechanisms involving key proteins associated with NDDs, specifically APP, MAPT, and SNCA. It outlines various regulators, their effects (either up- or down-regulation), and the mechanisms through which they exert their influence on the respective target proteins, as provided in Table 3. APP regulation is primarily driven by cleavage mechanisms from BACE1 and Gamma-secretase, alongside transcriptional regulation by CTCF. MAPT modulation is largely characterized by phosphorylation events mediated by GSK3B, CDK5, TTBK1, and DYRK1A, as well as dephosphorylation by PP2B. APOE upregulates the binding of MAPT. For SNCA, up-regulatory phosphorylation is mediated particularly by DYRKIA, and down-regulatory phosphorylation effects are observed, from kinases like LRRK2, GRK, SYK, and members of the PLK family, along with ubiquitination of PRKN.

**Table 3:** Regulatory interactions, their effects, and the mechanism of action on the target proteins APP, MAPT, and SNCA

Regulators	Effects (up/down regulation)	Mechanism of action	Target
BACE1	Up-regulates activity	Cleavage	APP
GAMMA SECRETASE	Up-regulates activity	Cleavage	APP
MAPK8/10	Up-regulates	Phosphorylation	APP
CDK5RA2	Upregulate quantity by stabilization	Phosphorylation	APP
PLD3	Up-regulates quantity	Binding	APP
PITRM1	Up-regulates activity	Cleavage	APP
ABCA7	Down-regulates quantity	Relocalization	APP
CTCF	Upregulates quantity by expression	Transcriptional regulation	APP
GSK3B	Down-regulates	Phosphorylation	MAPT
CDK5/CDK5R1	Down-regulates	Phosphorylation	MAPT
APOE	Up-regulates activity	Binding	MAPT
PP2B	Up-regulates	Dephosphorylation	MAPT
GSK3A	Down-regulates	Phosphorylation	MAPT
CAPN1	Down-regulates activity	Cleavage	MAPT
SGK1	Down-regulates	Phosphorylation	MAPT
CSNK1D	Down-regulates	Phosphorylation	MAPT
TTBK1	Down-regulates	Phosphorylation	MAPT
DYRK1A	Down-regulates	Phosphorylation	MAPT
SNCAIP	Up-regulates activity	Binding	SNCA
LRRK2	Down-regulates activity	Phosphorylation	SNCA
GRK5	Down-regulates activity	Phosphorylation	SNCA
DYRK1A	Up-regulates	Phosphorylation	SNCA
SYK	Down-regulates	Phosphorylation	SNCA
PLK1/2/3	Down-regulates activity	Phosphorylation	SNCA
PPP2CA/B	Down-regulates activity	Dephosphorylation	SNCA
GRK2	Down-regulates activity	Phosphorylation	SNCA
PRKN	Down-regulates by destabilization	Ubiquitination	SNCA

### Gene Ontology

**Fig. 2:**
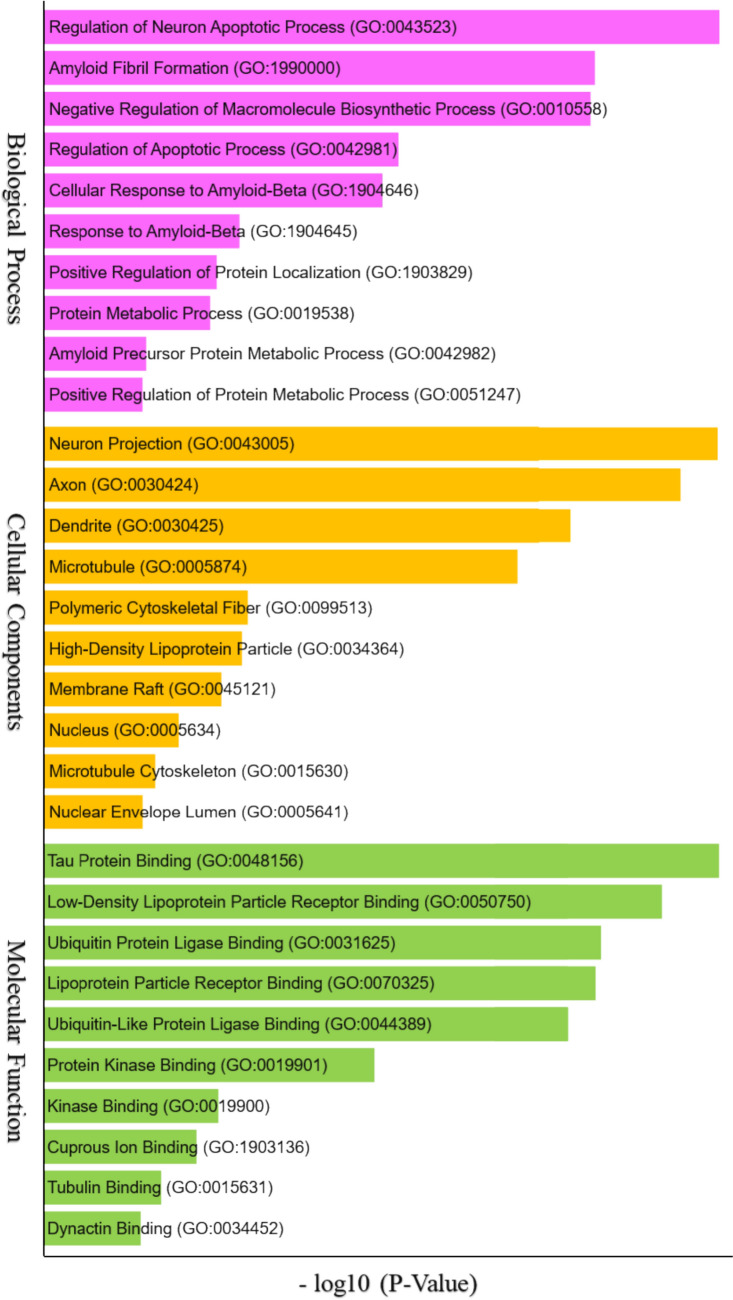
Gene ontology of top interactors with enriched terms BP, MF, and CC based on statistically significant FDR < 0.05 and nominal *p* value

The Gene Ontology (GO) analysis of the top functional interacting protein partners derived from each of the networks of APP, MAPT, and SNCA was performed using Enrichr and thus, FDR and *p*-adjusted values < 0.05 were retained as significantly enriched GO terms for the top protein interactomes as of with three important functional categories includes BP, MF, and CC. The top ten significantly GO enrichment terms of BP, CC, and MF were depicted in Fig. 2 with ontologies. The BP terms highlighted more specific biological programs such as the regulation of neuron apoptotic process, amyloid fibril formation, and cellular response to amyloid beta. Additionally, CC encompasses cellular entities such as neuron projections, axons, and dendrites, as well as microtubules. Finally, MF-enriched terms included the tau protein binding, ubiquitin protein ligase binding, and tau protein kinase activity.

### KEGG Pathway

**Fig. 3:**
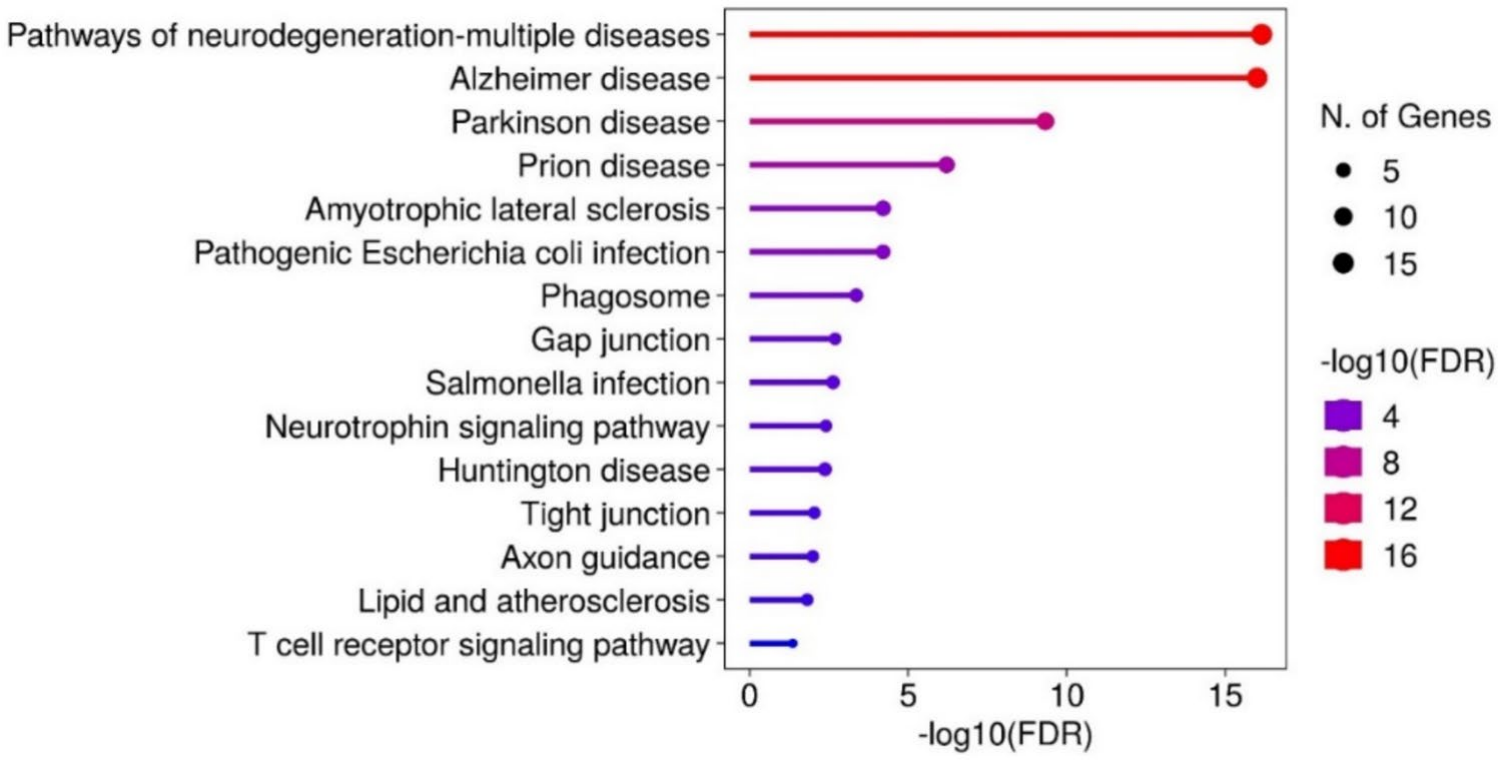
Pathway enrichment analysis of APP, MAPT, and SNCA interacting proteins, visualizing the top-enriched pathways and their statistical significance

**Fig. 4:**
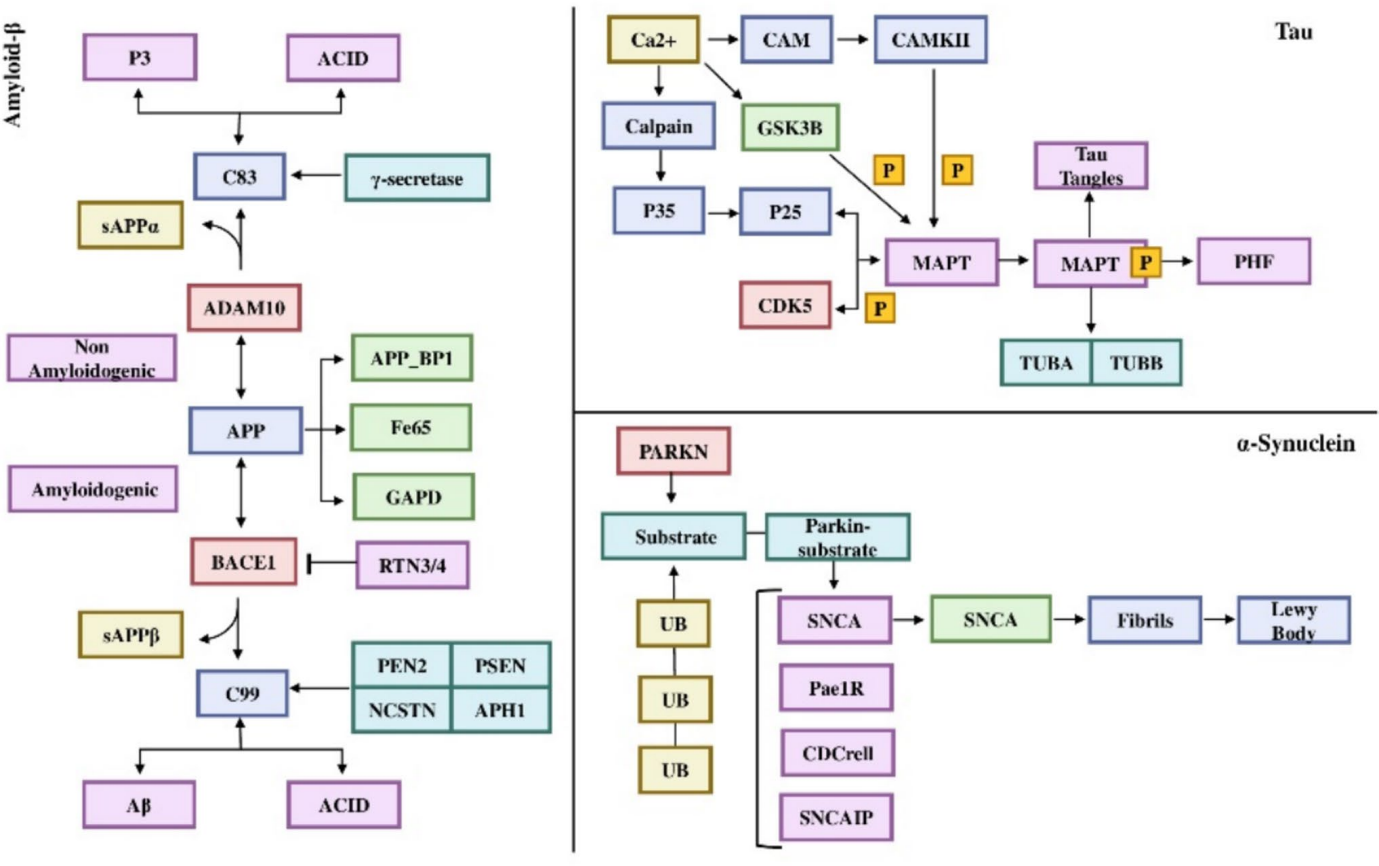
KEGG pathways of neurodegeneration—multiple diseases, including Alzheimer's and Parkinson's diseases, focusing on three critical protein systems: APP-amyloid-β, MAPT-tau, and SNCA-α-synuclein, which represent the core mechanisms of protein misfolding and aggregation that drive neurodegeneration

The pathway analysis further elucidated the functional pathways associated with the top interactors of APP, MAPT, and SNCA. Our analysis revealed the top 15 enrichment pathways, with notable distinctions in enrichment levels highlighted by the FDR, adjusted *p* value as depicted in Fig. 3. The pathways of neurodegeneration-multiple diseases (Fig. 4), including Alzheimer's disease and Parkinson's disease, exhibit the highest levels of enrichment and involve a large number of gene counts, suggesting a strong convergence of these interacting proteins on core neurodegenerative processes. Other significantly enriched pathways include prion disease, amyotrophic lateral sclerosis, and huntington disease. Neurotrophin signaling pathway, highlighting molecular mechanisms related to APP, MAPT, and SNCA interactors. The enrichment in pathways such as gap junction, axon guidance, and tight junction further emphasizes the role of synaptic dysfunction and impaired neuronal connectivity in neurodegeneration.

## Discussion

This study systematically mapped the interactomes of three central neurodegeneration-associated proteins- APP, MAPT, and SNCA, revealing their pivotal roles as initiators of protein aggregation, misfolding, and cross-seeding events. This aligns with established cascades of Aβ, tau, and α-syn pathologies in AD and PD brains and directly correlates with neurodegeneration. Aβ affects synaptic transmission; α-syn interferes with the import of mitochondrial proteins; and tau interferes with microtubule activity and neuronal transport processes (Sweeney et al. 2017; Sengupta and Kayed 2022). Importantly, existing research provides compelling evidence that tau, α-syn, and Aβ interact synergistically and promote the accumulation and aggregation of each other to induce cognitive decline (Clinton et al. 2010; Sengupta and Kayed 2022). Here, neuropathological and experimental findings, which together pinpoint interlinks between the aggregation of Aβ, Tau, and α-syn (Spires-Jones et al. 2017). The Aβ and tau interactomes within the consensus integrated network reveal fundamental distinctions in their biological roles, pathways of aggregation, and spatial–temporal progression in NDDs. Aβ network is primarily associated with immune response and metabolic stress, whereas the tau network is enriched in interactors that are more tightly linked to cytoskeletal dynamics and microtubule stability (Roda et al. 2022). α-syn protein network interactome of PD highlights their key roles for mitochondrial functionality, and the pathways of degradation (Tomkins and Manzoni 2021).

Protein interactors can interact to form a stable interface, but remarkably, only a few fractions of those that contribute to aggregation, may reveal attractive therapeutic targets in order to inhibit protein aggregation in NDD (Balasubramaniam et al. 2019). Protein interactome mapping identified 100 high-confidence partners regulating the biology of each seed protein-APP, MAPT, and SNCA, revealing distinct yet overlapping networks with significant PPI enrichment (*p* < 1.0e−16). The MAPT network exhibited the highest average node degree compared to the APP and SNCA networks. The top 10 interactors for each protein were identified with a high confidence score (≥ 0.9). APP interactome is associated with amyloidogenic regulators such as BACE1, APOE, and PSEN1 (Grewal et al. 2024; Biza et al. 2017). The MAPT interactome, clustered around tubulin subunits and kinases CDK5 and GSK3B, highlights its critical involvement in cytoskeletal dynamics and hyperphosphorylation characteristic of tau (Drummond et al. 2020). The SNCA interactome, featuring proteins like PRKN, PARK7, and SNCAIP, points to the complex mechanisms governing α-syn aggregation (Hernandez et al. 2020). These findings align with network medicine principles, where hub proteins and highly interconnected nodes often represent critical regulators of disease (Barabási et al. 2011; Chen et al. 2023). Hence, our interaction network, which recapitulates findings from prior studies, highly supported the functional relevance in neurodegeneration (Limviphuvadh et al. 2007; Hosp et al. 2015).

Hub proteins, the top 10 ranked with high-degree distribution and betweenness centrality, within each interactome, pinpoint key players with high network connectivity based on graph theory. APBB1, BACE1, and PSEN1 emerged as key hub nodes in the APP interactome, exhibiting the highest degree (≥ 7); GSK3B and CDK5 in the MAPT interactome; and PARK7 and LRRK2 in the SNCA network. Nodes with a ratio of centrality > 0.5 in accordance with existing studies were considered to be influential hub proteins within their respective networks as promising therapeutic targets (Ahmad et al. 2016). The integrated network comprises distinct nodes with edges, revealing nine protein interactors interacting with all seed proteins of APP, MAPT, and SNCA, found to be key mediators of Aβ, tau, and α-syn pathologies. Targeting these shared interactors, such as APOE, PRKN, and PSEN1, serves as an integrated therapeutic node that facilitates the development of multimodal therapies for diverse NDDs (Haenig et al. 2020; Calabrese et al. 2022). For instance, the convergence of shared nodes among three protein interactomes is consistent with recent network analysis based on molecular evidence to address the crosstalk of both AD-PD comorbidity (Hosp et al. 2015; Ruffini et al. 2022). The specific overlaps between pairs of interactomes further delineate the interconnectedness of these disease-related protein networks.

The Human Protein Atlas revealed direct interactors, along with their cell type/tissue specificity, and subcellular localization. For instance, the direct interactors of APP are BACE1, APBB1, SORL1, and PSEN1 play central roles in amyloid processing (Grewal et al. 2024). Similarly, the direct interaction of MAPT with GSK3B kinase reinforces tau phosphorylation (Drummond et al. 2020). The direct interactions of SNCA with SNCAIP and APOE modulate α-syn (Hernandez et al. 2020). All these interactors are primarily expressed in the brain, with neuronal cell types, predominantly localized from endomembrane system to cytoplasm and nucleus. This is consistent with single-cell transcriptomic studies linking NDD-associated proteins supporting cell-type susceptibility observed in AD and PD (Mathys et al. 2019; Das et al. 2024). Disease-gene association provided strong associations of specific protein-encoding genes with AD and PD. The top hundred genes were prioritized based on GDA scores validated from multiple sources. Notably, gene encoded proteins like APP, MAPT, PSEN1, APOE ε4 allele, BACE1, and TREM2 are strongly associated with AD (H. Zhang et al. 2021a, b; Soler-López et al. 2011), and SNCA, PINK1, LRRK2, PARKN, PARK7, and MAOB, linked to PD (Va acute zquez-Ve acute lez and Zoghbi 2021). These protein-encoding genes have a distinct regulatory role in disease progression and are widely recognized as a genetic risk factor for AD-PD, supported by extensive evidence and EI (Evidence Index) values > 0.9, with a GDA score of 1 and DSI ≥ 0.5, are considered the most associated target genes/proteins, which cause early and late-onset and progression of NDD (Haenig et al. 2020; Zhang et al. 2021a; Karimi-Moghadam et al. 2018).

Regulatory factors regulating the mechanisms of APP, MAPT, and SNCA highlighted key interactors, for instance, BACE1, PSEN1 (presenilin)/NCSTN (nicastrin) of gamma-secretase upregulate the protein cleavage of APP, resulting in Aβ42, a major component of amyloid plaques. GSK3B, CDK5, and DYRKIA regulated phosphorylation of MAPT (Priya et al. 2021). APOE's direct binding interaction with MAPT highlights its role in disease pathology. LRRK2 down-regulates the phosphorylation of SNCA, while PRKN ubiquitination of SNCA enhances clearance. These insights emphasize the importance of targeting upstream regulatory pathways to modulate seed protein pathology. GO analysis of top interacting protein partners, over-represented by top 10 significantly enriched ontology terms with statistically significant *p* value and FDR of < 0.05, describing BP, MF, and CC, and is highly correlated with the existing research. BP notably included positive/negative regulation of neuronal death (apoptosis), and cellular response to amyloid beta. CC category captured cellular location like neuron projection, axons, and dendrites. MF represents molecular-level activities, such as amyloid-beta binding, tau protein binding, ubiquitin-proteosome system, and kinase activity. KEGG pathway maps, specifically “pathways of neurodegeneration-multiple disease,” is the most enriched pathway with a large count of interacting partners shared by APP, MAPT, and SNCA interactors, in the etiology of diverse neuropathologies related to AD-PD seed proteins, and are correlated with current knowledge of the biological pathways implicated in NDD (Zhang et al. 2021b; Xu et al. 2023).

Protein interactors and network hubs in our study necessitate laboratory-based validation. This is a general challenge for computational studies, which emphasizes that experimental validation is essential for confirming physical interactions, functional roles, and biological relevance of identified interactions, especially those mediating AD-PD, particularly the shared interactors across multiple disease proteins, which would strengthen their relevance as therapeutic targets (Milano et al. 2022). We have now explicitly stated this limitation and further encourage future experimental studies to validate these predicted interactions in a disease-relevant cellular model. Further, it is essential to integrate multi-omics profiles to define the interrelations between protein pathways that underlie the tau, α-syn, and Aβ cascades linked to the progression of NDDs. Future therapeutic strategies that target multiple aspects of disease-related factors or identify a key protein that is interlinked with all disease-driving pathways hold a promising approach (Sweeney et al. 2017). Additionally, exploring the impact of genetic variants on these interactomes could enhance personalized medicine approaches. These interactomes highlight the limitations of single-target therapies and promote multi-targeted strategies, providing a framework for multi-targeted therapies (Sengupta and Kayed 2022).

## Conclusion

Interactomes of three key proteins—APP, MAPT, and SNCA were investigated through network analysis and identified significant protein interactions. Molecular aspects of the Aβ-tau-αsyn protein interactome analysis and functional protein associations underscore the biological significance of their protein interactors in mediating protein aggregation, misfolding, and pathological cross-seeding events, which are central to the progressive development of NDDs like AD and PD. The protein interactomes of Aβ, α-syn, and tau are defined by both direct binding events and indirect regulatory relationships that modulate their pathological aggregation, neurotoxicity, and clearance. Several protein interactors have been identified as key regulators of protein aggregation, including cleaving enzymes, phosphorylation-dependent kinases, transcriptional and translational regulators, and enzyme transporters. Furthermore, the research explored direct interactors, hub proteins, shared partners, regulatory mechanisms, genetic modifiers, enriched gene ontologies, functional pathways, and associated factors, underscoring the potential for developing targeted therapies that disrupt pathological protein aggregation and propagation. Additionally, we revealed that mechanistic crosstalk between AD and PD is interlinked with their pathogenic proteins Aβ, tau, and α-syn. All together provide valuable insights into key roles in disease mechanisms and potential therapeutic targets for drug development and effective disease-modifying therapies, potentially even more preventive strategies. Future research on the identification of novel targeted therapeutic interventions for multiple disease aspects states that targeting direct interactors can prevent the progression of these devastating NDDs.

## Data Availability

Any request regarding the dataset supporting the conclusions of this article should be proposed to the authors.
